# Understanding scientists’ communication challenges at the intersection of climate and agriculture

**DOI:** 10.1371/journal.pone.0269927

**Published:** 2022-08-02

**Authors:** Jackie M. Getson, Sarah P. Church, Brennan G. Radulski, Anders E. Sjöstrand, Junyu Lu, Linda S. Prokopy

**Affiliations:** Department of Forestry and Natural Resources, Purdue University, West Lafayette, Indiana, United States of America; Soil and Water Resources Institute ELGO-DIMITRA, GREECE

## Abstract

In the United States, a public debate remains about the existence and effects of anthropogenic climate change. This skepticism is present in the agricultural sector, rendering climate science communication challenging. Due to the polarization of climate change issues and the concurrent need for agricultural adaptation, we sought to examine how scientists communicate in this sector. A survey, administered to climate scientists and pertinent U.S. federal agency staff (response rate = 43%), was conducted to examine perspectives on communicating with five agricultural stakeholder groups: agribusinesses, crop advisors, general public, producers, and policymakers. We focused on three aspects of the communication process with these stakeholders to evaluate if scientists, as messengers, were following best practices–communicator training, knowledge of stakeholder, and terminology use. We found scientists valued communication training; however, the majority had not attended formal training. Scientists had different views on climate change than producers and crop advisors but understood their perspective and were deliberate with their communication with different audiences. This suggests stakeholder knowledge and terminology use do not hinder communication between scientist and stakeholder. We also highlight three communication challenges present across stakeholder groups–stakeholder knowledge, timescale, and scientific uncertainty–and others that were specific to each stakeholder group. Future research should support scientists by identifying and resolving barriers to training and effective communication strategies for each stakeholder group that addresses these challenges.

## Introduction

The agricultural sector in the United States (U.S.) is facing threats from uncertainty and risk associated with climate change [1]. The implications of potential risk include reduced productivity of crops and livestock, soil and water quality and quantity issues, increased pest and disease issues, and economic hardship [2, 3]. While the Intergovernmental Panel on Climate Change (IPCC) Six Assessment Report [4] details these potential risks associated with climate change, there remains debate around the existence of anthropogenic climate change in the U.S. [5]. Prior research indicates there is varying belief in anthropogenic climate change among stakeholders in the agricultural sector. In a study conducted in California, approximately half (54%) of surveyed producers (i.e., farmers) agreed the global climate is changing, with 35% believing human activities played a role in causing climate change [6]. In the Midwest, it was found that only 8% of producers believed climate change is mostly anthropogenic, with 33% believing it is equally human and naturally occurring, and 25% believing it is mostly naturally occurring [7]. Meanwhile, 5% of certified crop advisors/agricultural retailers believed climate change is mostly anthropogenic, with 34% believing it is equally human and naturally occurring, and 30% believing it is mostly naturally occurring [8], More recent research reflects changes in agricultural stakeholders’ beliefs about climate change. There is an increasing acknowledgement in the occurrence of climate change among producers in the U.S. [9–12], which sometimes is associated with producer exposure to extreme weather events [9, 13, 14], although this is not always the case [10, 15]). This mismatch in producer and agricultural advisor climate change beliefs could create challenges in how to communicate climate science [8]. Moreover, producers who believe in anthropogenic climate change are more likely to support adaptive or mitigative farm practices [7]. All of these complexities feed into considerations of climate science communication.

One cause of the gap between climate scientists’ understanding of, and stakeholders’ belief in, climate science is due to how the science is communicated [16]; poor communication strategies, particularly regarding risk communication, can lead to increased dispersal of climate change disinformation, which can influence disbelief in climate science [17]. Likewise, in the agricultural sector, scientists’ perceptions about their own communication roles and strategies may be impeding this sector’s ability to adapt to and/or mitigate the impacts of climate change because of their perceived role as objective scientists who present but do not interpret climate science information [18]. The agricultural sector is comprised of multiple stakeholder groups in addition to producers. How producers make decisions and find information is complex; for example, they use many sources of information for farm management decisions, including family, other producers, media sources, university and governmental sources, and crop advisors [19, 20]. Considering the complexity of decision-making and multitude of information sources, we sought to answer the following questions: How do climate scientists communicate with agricultural stakeholder groups about climate science? What are climate scientists’ current challenges with climate change communication to different agricultural stakeholder groups?

In this study, we conducted an exploratory study to examine scientists’ perspectives on their communication with five agricultural stakeholder groups: agribusiness, crop advisors, general public, crop/livestock producers (i.e., farmers), and policymakers. We used an online survey instrument to query scientists and professionals in the agroclimate field to evaluate if: (1) scientists participated in formal communication training, (2) scientists understood agricultural stakeholder groups’ perception of climate science, (3) scientist terminology use was framed based on target stakeholder group, and (4) there were different communication challenges by stakeholder group. Broadly, our results show although it may not be their formal responsibility and although they may lack training, some scientists understand stakeholder perceptions and modify their terminology based on the group they are talking to. We discuss what we can learn from these successful communication efforts to further support scientists’ communication efforts.

## Background

### Messenger and knowledge deficit model

As messengers, climate scientists have long engaged in communications and outreach with public audiences, serving as direct sources of scientific information. More scientists are interacting with public audiences both directly and indirectly through the mass media. A survey of 3,748 U.S.-based scientists connected to the American Association for the Advancement of science found almost all (98%) scientists have interacted with citizens from time to time and over half (51%) have communicated research findings with reporters [21]. Although more scientists are making efforts to communicate with public audiences and the media through various modes of communication [22], not all scientists are experts in the science behind effective communication [23]. This becomes apparent when exploring models of communication between scientists (information sources or messengers) and the public (intended audience or recipients of the message). Science communication has previously been understood as a one-way flow of information from a source to an audience, where communicators assume audiences are ignorant to science issues–the knowledge deficit model. However, research suggests effective communication of science is more complex, with many different social factors impacting an audience’s receptiveness of scientific information [24]. Effective communication also requires methods grounded in social science research [25–27], some of which has resulted in newer understandings of the transfer of information between sources and intended audiences (e.g., a model of public engagement [28]). Despite this evidence, many scientists still defer to the knowledge deficit model of communication [29], implying some may not always be strategic about their communication. In the context of climate scientists as messengers of climate change science to agricultural stakeholders, utilizing the knowledge deficit model to inform communication may negatively impact efforts to move agricultural stakeholders towards a consensus on the occurrence of climate change and its causes, as communication attempts designed to increase knowledge and gain support for a science issue tend to be ineffective [30].

### Science communication training

Some critics have suggested scientists should only conduct research [31]; however, scientists are key actors in the communication of their research to lay audiences [26], are encouraged to increase their presence in the public sphere [32] and increasing evidence suggests scientists see themselves in an advocacy role [22]. Recommendations to become an effective communicator are often distilled into three main messages: participate in formal communication training, understand the audience, and frame the message appropriately [26, 33–35]. Communication training is important for scientists since training can enhance successful communication between scientists and stakeholder groups [36]. Scientists should be trained in how to communicate their science to the public, and that, in general, scientists do not receive formal science communication training [34, 35]. In 2013, 24 out of the 27 climate change adaptation plans from U.S. federal agencies identified a need for communication research and training for scientists [37]. Specifically, communication training should be targeted at climate scientists (or scientists doing climate-related work) to promote successful communication [20, 38].

### Understanding an audience

Effective communication requires the information provider understand what information the audience needs to inform decision-making; a substantial part of this is understanding what the audience already knows [39] in order to implement an effective communication strategy [40, 41]. However, the complexity of climate science compounds communication challenges as it is a difficult topic to communicate to nonscientists [42, 43]. There are multiple factors to consider to know an audience. Climate change communication entails risk communication, as a changing climate [43, 44] contributes to risks such as increased extremes in drought, heat, and precipitation and subsequent impacts [45]. People conceive of risks differently, depending on many factors such as social networks, education, values, and more [26, 46, 47]. One important aspect of communication broadly is to identify trusted messengers to deliver appropriate messages, whether that be scientists, the media, family, neighbors, or other trusted advisors [19, 47]. Trusted messengers are especially important when linked to perceptions of risk, like climate change risk, as people look to these trusted sources to aid in decision-making when the conditions of the risk are uncertain, or they do not have the knowledge to feel confident in their decision-making [48]. Indeed, the perceived credibility of a communicator can influence an audience’s interpretation of and belief in science, such that higher levels of perceived credibility can increase the persuasiveness or acceptance of science messages [49, 50]. Bolsen, Palm, and Kingsland [51] found when messages were attributed to climate scientists, the messenger attribution had no or a negative effect on the audiences’ belief of the message (be it about national security, climate change, and/or scientific consensus on climate change). In addition to identifying trusted messengers, knowing an audience entails understanding how that audience perceives risk; for example, how susceptible a particular audience thinks they are to a threat and how well they can cope with that threat [52]. Overall, knowing social factors that may influence an audience’s perceptions and opinions about science, as well people’s perceptions of the threat as credible and their ability to act effectively, can aid in framing relevant messages about science facts to the audience [26, 53].

### Message framing

Message frames are used in communication to make complex science (such as climate change) and associated issues more comprehensible, relatable, and valued by lay audiences. This is done by putting emphasis on certain interpretations, concepts, and/or storylines depending on the audience to which information is communicated [54, 55]. There are a variety of framing techniques that can be utilized in communication [26, 56–59]. For example, Dickinson et al. [56] found birdwatchers were more interested in taking personal action on climate change when presented with a framing statement about danger to birds from climate change than when presented with a framing statement about danger to humans from climate change. This example frames climate change in a manner that resonates more with a particular lay audience [57], by linking audience values to issues of climate change. Similarly, terms or concepts associated with science issues may have multiple understood meanings that lead to confusion [60]. Terms with multiple meanings have the ability to impact stakeholder perceptions, beliefs, and understanding of science issues differently. Particular to climate change communication, many studies highlight misinterpretations of certain terms and concepts [61–65]. For example, Howe et al. [66] illustrated the complications of explaining uncertainty to the public. Climate science often uses uncertainty to represent statistical probabilities, scientific confidence levels, model predictions, and possible risks due to the degree of warming, level of emissions, and climate sensitivity to produced emissions. However, the public interpret uncertainty as if the experts do not know something will happen; uncertainty about climate change may mean that it will not happen at all [62]. Likewise, where a scientist may use the words “positive” or “negative” to indicate the direction of a relationship, nonscientific audiences may interpret these terms meaning “good” and “bad,” respectively [67]. The way of framing climate change, and the choice of terms when doing so, is therefore highly important due to its influence on audience interpretation.

### Communication challenges with producers

Different stakeholder groups have varying beliefs about climate change [7, 19, 57, 68]. Wilke and Morton [61] found climate scientists, knowing the conservative nature of producers and the political contention around climate change, try to distance their messages about climate change risk from what may be considered a political agenda in order to remain neutral and build trust. Additionally, scientists attempted to frame climate change information such that producers may perceive it as relevant and important (e.g., providing producers with risk assessments [61]). Making climate change relevant, or personal, in this case can aid in avoiding the controversies and political connotations around climate change [61], and in establishing climate scientists as credible sources for climate change information [49].

Similarly, temporal differences in immediate versus long-term climate change effects make communicating climate science a challenge because climate change effects are often not immediately noticeable and visible, or even geographically present [33]. Terminology use may be particularly sensitive to different agricultural audiences. Wilke [69] suggested not using the term “global warming” with agricultural audiences, because “warming” is only one aspect of global climate change. Instead, using the holistic term “climate change” in conversations may influence audiences’ acceptance of climate science information as well as aiding in belief of anthropogenic climate change [69]. Effective communication to agricultural audiences is perceived to be greatly influenced by complex social factors like the political atmosphere; therefore, some recommend keeping climate science and politics separate [61]. Disinformation campaigns like those propagated by ExxonMobil [70] and the mainstream media’s balanced presentation of perspectives from climate change scientists and climate change contrarians, allowing them equal or in some cases more air time to contrarians despite the majority of scientists accept climate change [32], may reinforce a belief in the uncertainty of climate change impacts causing a “wait and see” stance for many producers [71].

## Methods

### Scientist survey

Targeted participants were gathered from two sources. The first was scientists identified through a previous survey as a climate scientist that worked on a climate and agriculture-related project funded by the United States Department of Agriculture National Institute of Food and Agriculture (USDA-NIFA) [72] (henceforth referred to as the climate portfolio; N = 301). The second was an internet search to identify government agencies that manage or conduct work related to climate issues. The following organizations were contacted to complete the survey: American Association of State Climatologists (AASC), Environmental Protection Agency (EPA) Agriculture Center Regional Agriculture Advisors, Landscape Conservation Cooperative (LCC) network staff, National Oceanic and Atmospheric Administration (NOAA) regional climate centers, USDA Climate Hubs and Forest Service of Sustainability and Climate Change, and United States Geological Survey (USGS) Climate Science Centers Directors. Because this survey focused on communication and outreach, all job roles were included from relevant office staff who were scientists working on different climate-related issues and/or interacting with stakeholders, through directors to research leaders (N = 339). The project advisory committee piloted the survey prior to distribution but their results were not included in this analysis. The advisory committee was comprised of faculty conducting research in climate and agriculture and/or with expertise in extension working with stakeholders, and directors of climate centers. The NIFA National Program Leader for Agroclimatology announced the survey via email on March 28, 2017. The initial invitation was sent March 30 to complete the questionnaire using the online survey platform Qualtrics; in total, three reminders were sent out only to those that had not responded prior to closing on June 2, 2017. Both surveys were reviewed and approved by the Purdue University Institutional Review Board. The protocol numbers are 1512016847 and 1702018843, respectively.

Questions were developed to capture scientists’ perspectives on their: experience with climate science and with the five agricultural stakeholders, education and communications training, knowledge of stakeholders, terminology use with stakeholders, and stakeholder communication challenges. To identify the terms to measure, a literature analysis was conducted for recommended terms to use or avoid [61, 63, 64, 67, 73–76]. The five stakeholder groups explored were: agribusinesses, crop advisors, crop and/or livestock producers, general public, and policymakers. Using a bipolar 5-point scale from “strongly disagree” to “strongly agree” we assessed the scientists’ views on the current state of climate change and agroecosystems science as well as their professional roles and responsibilities. We then gathered scientists’ views on climate science through a modified block of statements originally developed to assess producers’ views on climate change using a bipolar 5-point scale from “strongly disagree” to “strongly agree” [77]. Additionally, these statements were used to ascertain scientists’ perspectives on how five stakeholder groups would respond to the same questions. Respondents that worked with multiple stakeholders were asked to select the stakeholder that was their highest priority and then asked the series of questions regarding that stakeholder group. These respondents were then provided the opportunity to answer the same block of questions for additional stakeholders. In addition to climate change perspectives, we asked scientists to describe challenges in communicating climate change issues to each stakeholder group. The entirety of the survey is included in the S1 File.

### Producer and crop advisor surveys

To compare scientists’ perspective to stakeholders, data from two additional surveys were used. Both surveys were conducted during 2016 in the U.S. Midwest, which consists of 12 states. The producer survey was conducted from June to October (N = 2633) [77] and the crop advisor survey was conducted from November to December (N = 2719) [78]. In this study, crop advisors were defined as respondents whose primary occupation was an agricultural advisor, respondents that provide advice to corn producers, and/or respondents that educate or train other advisors.

### Analysis

Prior to analysis, the dataset was cleaned to confirm responses fit within the correct datatype and to correct any typographical errors in open responses. For open ended responses, one researcher developed the initial codebook by coding a portion of the responses into prevalent themes. Then, two additional researchers used the initial codebook to independently code each of the previously coded open responses. The research team then met to discuss and resolve coding discrepancies. Through these discussions, the codebook was finalized to the agreement of the research team [79]. One researcher then reconciled coding discrepancies identified through the codebook development process. All codes were ultimately agreed upon by all three researchers.

Statistical analyses were conducted in R (version 3.5.1). Since the responses were not normally distributed (determined by a Shapiro-Wilk normality test), a non-parametric Kruskal-Wallis test by ranks was used to determine if the distribution of responses were significantly different across stakeholders overall by question. To determine if there was a significant difference between two stakeholders by question, a Wilcoxon-Mann-Whitney test with Bonferroni correction method adjusting for family-wise error rate was employed to conduct pairwise comparison.

## Results

### Response demographics

The overall scientist survey response rate was 42.8% (N = 265). The intent of the initial survey was to collect perspective from all roles within organizations; however, respondents were primarily scientists. Only eight respondents identified as nonscientists and these were removed from analysis and response rate calculation. Surveys were considered complete if they answered at least one question (S1 Table). Not all respondents answered all questions; therefore, response rates vary by question (“N” henceforth indicates the total number of respondents). Each of the contacted organizations (government/AACS) had at least one respondent, with the majority of responses from the USDA, with the following distribution: AASC = 9.5% (n = 10), EPA = 1.0% (n = 1), LCC = 27.6% (n = 29), NOAA = 12.4% (n = 13), USDA = 33.3% (n = 35), USGS = 16.2% (n = 17). The following results are presented as a combined audience since no statistical differences were found when aggregated into separate groups. Mean respondent age was 52.5 ± 10.6 *sd* (N = 216) and were majority male (74.4%, N = 215). Respondents most frequently identified as a life, climate, and/or physical scientist (S1 Fig); since participants were not all climate scientists by education/degree but conducted climate-related work, respondents are not referred to as “climate scientists” but rather “scientists.”

### Experience

The respondents indicated being in their current role for 11.6 ± 10.1 *sd* (N = 220) years and spent on average 16.9 ± 11.6 *sd* years (N = 218) in a climate-related field as their occupation. The majority (86.4%; N = 213) worked with two or more stakeholder groups, most commonly the “general public” (S2 Fig). The respondents that indicated experience with multiple stakeholder groups were queried on the group they selected as their main priority. Of those, most (88.9%; N = 181) indicated their focus was “producers,” “general public,” or “policymakers.”

### Communication training

Nearly all (90.7%) of the respondents (N = 225) agreed that scientists should receive training on how to communicate scientific findings to nonscientists. However, the majority (64.9%; N = 225) were not formally trained on how to communicate with stakeholders. Open ended responses were coded for formal and informal training in which they had participated; these included formal training (N = 74): “workshops” (n = 28), “classes” (n = 11), and “in-service” (n = 11) and informal (N = 162): “on-the-job” (n = 101) and “publications” (n = 31).

### Perception of stakeholders

Scientists ranked their level of agreement with each climate change statement. They were also asked to rate their perspective on how the five stakeholders would respond to the same climate change statements. All but one statement (“Earth’s climate conditions occur in a cyclical pattern”) was found to have responses that were statistically different across the stakeholder groups (S2 Table; *p-value*). The scientists responded that all stakeholder groups, relative to their own perspectives, would be significantly more likely to agree with two statements “Earth’s climate conditions occur at random with no cycles or trends” and “Even if climate changes, we cannot predict what those changes will be in the future.” The scientists responded that all stakeholder groups would be significantly less likely to agree to four statements: “Climate change is happening,” “Human activities are contributing to climate change,” and “Human activities are the primary driver of climate change,” and “There is enough evidence that climate is changing.” The scientists thought there would be significantly more agreement by producers, public, and policymakers, with the statement regarding climate change not affecting how they live/operate. Lastly, the scientists perceived producers and policymakers were significantly more likely to agree that they as a group would distrust scientists working on climate-related issues than a general audience (Table 1).

**Table 1:** Scientists’ agreement to statements compared to scientists’ perspective on how stakeholders would respond to the same statements regarding climate change.

Statement	Scientists		Agribusinesses		Crop Advisors		Producers		Public		Policymakers	
	*n*	*M*	*n*	*ΔM*	*n*	*ΔM*	*n*	*ΔM*	*n*	*ΔM*	*n*	*ΔM*
Earth’s climate conditions occur at random with no cycles or trends.	245	1.4	21	+1.0***	10	+0.9*	75	+1.3***	71	+1.4***	63	+1.1***
Earth’s climate conditions occur in a cyclical pattern.	243	3.5	21	+0.2	10	+0.3	74	+0.1	71	0	63	+0.2
Even if climate changes, we cannot predict what those changes will be in the future.	243	2.1	21	+1.2***	10	+0.8*	75	+1.3***	71	+1.1***	63	+1.0***
Climate change is happening.	242	4.7	21	-0.7***	10	-0.9***	75	-1.1***	71	-0.8***	62	-0.9***
Earth’s climate always changes.	242	4.1	21	-0.1	10	0	75	-0.2	71	-0.2*	63	-0.1
Human activities are contributing to climate change.	242	4.7	21	-1.2***	10	-1.1***	75	-1.5***	71	-1.0***	63	-1.2***
Human activities are the primary driver of climate change.	242	4.2	21	-1.2***	10	-1.1*	75	-1.6***	71	-0.9***	63	-1.3***
Climate change will not affect the way that the [stakeholders] [operate/lives].	242	1.7	21	+0.3	10	+0.6	75	+0.6***	71	+1.2***	63	+1.2***
There is enough evidence that climate is changing.	242	4.4	21	-0.7***	10	-0.9**	75	-1.2***	71	-1.0***	63	-1.1***
[Stakeholder] distrust scientists that work on climate-related issues.	242	2.5	21	+0.2	10	+0.1	75	+0.6***	71	+0.3	63	+0.4*

Notes. Survey questions: “Please indicate your level of agreement with the following statements” and “In your opinion, what would the majority of [stakeholder’s] level of agreement be with the following statements?” Two statements were rephrased to be specific to each stakeholder (indicated with brackets). All stakeholder group statements displayed “operate” except the general public. The statements that asked about the scientists’ own perspective were “Climate change will not affect the way that I live” and “The majority of the public distrusts scientists that work on climate related issues.”

scale: 1 = strongly disagree, 2 = disagree, 3 = neither agree nor disagree, 4 = agree, 5 = strongly agree

"p" column (Wilcoxon-Mann-Whitney test (two-sided) adjusted by Bonferroni correction method for multiple comparison) indicates the statistical significance of the climate scientists’ agreement with each climate statement comparing the climate scientists’ perspective of how the stakeholders would respond to the same climate statement; *, **, and *** indicate a statistical significance level at 0.05, 0.01, and 0.001 respectively. The p-value column (Kruskal-Wallis test) indicates whether the distribution of the responses for all six groups were different overall by statement.

### Knowledge of stakeholders

Additionally, eight of the climate change statements were asked to two stakeholder groups: producers (N = 2633) [77] and crop advisors (N = 2719) [78] on separate surveys conducted in 2016. The scientists’ own perspectives were compared to the actual perspectives for these two stakeholder groups. All statement responses were statistically different across all groups (S3 Table; *p-value*). Scientist responses were statistically different from producers and crop advisors across seven statements (exception “Earth’s climate conditions occur in cyclical pattern”). Producer and crop advisor responses were all statistically different from each other by statement (S3 Table; *p*).

**Fig 1:**
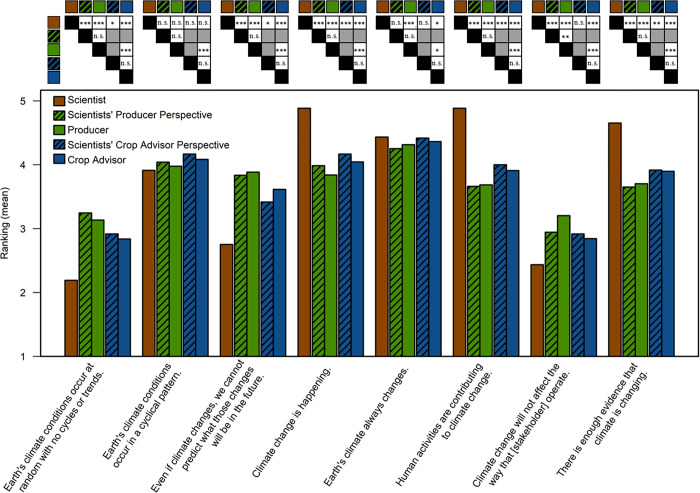
Scientists’ agreement with and perspectives on climate statements by producer and crop advisor compared to producer and crop advisor agreement. Significant relationships are demonstrated in tables above each statement (S2–S4 Tables). Gray indicates where pairwise comparisons were not applicable. Statistical significance is indicated by *, **, and ***, which corresponds to a statistical significance level at 0.05, 0.01, 0.001, respectively as well as no significance by “n.s.” The ranking scale was from 1–5, where: 1 = strongly disagree, 2 = disagree, 3 = neither agree nor disagree, 4 = agree, and 5 = strongly agree. Scientist perspectives were measured in a 2017 survey while surveys of producers (Singh et al. 2018) and crop advisors (Koundinya et al. 2017) were conducted as separately in 2016.

Finally, the scientists’ perspectives on each stakeholder group were compared to the actual perspectives of the two stakeholder groups. One statement was significantly different between the scientists’ producer perspective and the actual responses of the producers (Fig 1; S4 Table; *p-value*); however, this resulted in a mean ranking difference for “Climate change will not affect the way that producers operate” of +0.3. There were no significant differences between the scientists’ crop advisor perspective and the actual crop advisor responses (Fig 1, S4 Table; *p-value*).

### Framing

**Fig 2:**
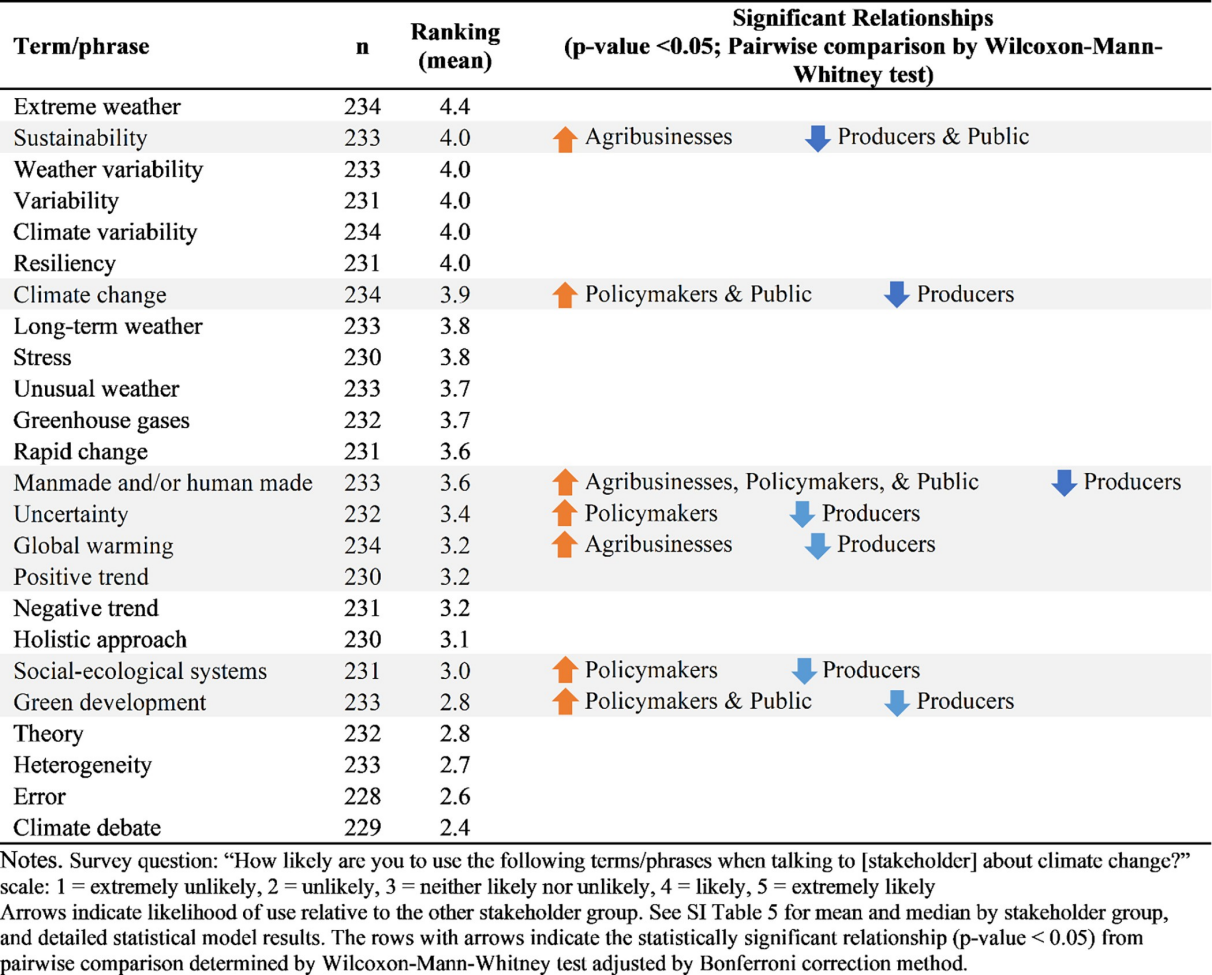
Scientists’ likelihood of using terminology sorted by overall decreasing likelihood ranking. Notes. Survey question: “How likely are you to use the following terms/phrases when talking to [stakeholder] about climate change?”. scale: 1 = extremely unlikely, 2 = unlikely, 3 = neither likely nor unlikely, 4 = likely, 5 = extremely likely Arrows indicate likelihood of use relative to the other stakeholder group. See S5 Table for mean and median by stakeholder group, and detailed statistical model results. The rows with arrows indicate the statistically significant relationship (p-value < 0.05) from pairwise comparison determined by Wilcoxon-Mann-Whitney test adjusted by Bonferroni correction method.

Terminology use is dependent on which stakeholder groups are being addressed for some of the terms (Fig 2; S5 Table). Overall, however, “extreme weather,” “weather variability,” “climate variability,” and “climate change,” were more likely to be used than the phrases “global warming” and “climate debate” for all stakeholder groups. Scientists were statistically significantly more likely to use “sustainability” with agribusinesses than with producers or the general public. They were statistically significantly more likely to use the phrases “climate change” and “green development” with policymakers and the general public than with producers. Using the term “manmade and/or human made” was statistically significantly more likely with agribusinesses, policymakers, and the general public than with producers. “Uncertainty” and “social-ecological systems” was statistically significantly more likely to be used with policymakers than producers. It was statistically significantly more likely to use “global warming” with agribusinesses than with producers and the general public, respectively.

### Challenges

We asked respondents to describe climate change communication challenges through an open-ended question in this survey. A total of 22 codes emerged from the challenges associated with communicating with stakeholders (Table 2; S6 Table includes representative quotes for each code or theme). The codes ranged from terminology choice (using layperson language), economic implications, making climate change personal to the stakeholders, accessibility issues (finding the right people, dissemination method, and time to meet), trust in the scientist, as well as group dynamics like belief systems (culture) and politics. The challenges were also variable across stakeholder groups with only three (stakeholder knowledge, timescale, and uncertainty) present for all five stakeholders (Table 2). These common challenges across stakeholder groups are highlighted below.

**Table 2:** Communication challenges by stakeholder group.

Challenge	Agribusinesses	Crop Advisors	Producers	General Public	Policymakers
	(n = 20)	(n = 10)	(n = 69)	(n = 65)	(n = 58)
Stakeholder knowledge	✓	**✓**	**✓**	**✓**	**✓**
Timescale	**✓**	**✓**	**✓**	**✓**	**✓**
Uncertainty	**✓**	**✓**	**✓**	**✓**	**✓**
Culture	**✓**		**✓**	**✓**	**✓**
Economics	**✓**	**✓**	**✓**		**✓**
Making it personal	**✓**		**✓**	**✓**	**✓**
Pathway of information		**✓**	**✓**	**✓**	**✓**
Politics	**✓**		**✓**	**✓**	**✓**
Trust		**✓**	**✓**	**✓**	**✓**
Using layperson language	**✓**		**✓**	**✓**	**✓**
Disinformation			**✓**	**✓**	**✓**
Pipeline to right people		**✓**	**✓**	**✓**	
Variability		**✓**	**✓**	**✓**	
Access to stakeholder			**✓**		**✓**
Collective strategy				**✓**	**✓**
None	**✓**		**✓**		
Overworked		**✓**	**✓**		
Scientist knowledge		**✓**	**✓**		
Fear of regulation			**✓**		
Knowledge gap			**✓**		
Risk management			**✓**		
Time management					**✓**

Notes. Survey question: “In your opinion, what is the fundamental challenge in communicating climate change issues to [stakeholder]?” A representative quote for each code is supplied in S6 Table. The checkmark indicates at least one respondents’ answer was coded for the theme.

Scientists reported communicating with their stakeholder groups can be a challenge because stakeholders’ scientific knowledge/training can be limited. Many scientists listed these issues for the general public, with a few listing them for each of the other stakeholder groups.

“*Most people have very little science training and remember little about what they have been taught. They do have a strong inherent interest about science; hence, the broad audiences of many public outlets about science*.”

“*Education is the key to make the public more aware of the issues and understand the sources. Public needs to be educated enough to question and understand the motives of the policymakers and politicians*.”

Stakeholder groups have difficulty conceptualizing the timescale of climate change. Thus, scientists reported challenges in helping their stakeholders think in terms of long-term trends rather than in the number of years needed for decision making. This theme was coded most frequently for all stakeholder groups except the general public.

“*Getting people to think of climate change and variability (including the past) on time scales of decades rather than years*.”

“*Short term business decisions (1–5 years) vs. climate projections (30–100 years)*.”

Scientists highlighted difficulties discussing uncertainty of timing and magnitude of climate change, but not the certainty of climate change. Many scientists noted this specifically in regards to communicating with policymakers, one noting that this is similar to how policymakers already deal with uncertainty in other areas of decision making. A few wrote that uncertainty is a challenge in communicating with producers.

“*Communicating uncertainties [is a challenge] honestly but in a context of all the other uncertainties within which policymakers already routinely make decisions, i.e., climate change uncertainty is not fundamentally different than many other uncertainties that already are accommodated, like drought risks, market fluctuations, and technological change. All are certain to come and we even have a pretty good idea of time scales and directions of change; none can be predicted precisely in either timing or magnitude; and proactive planning and accommodation are the most economical responses to each of these sources of uncertainty and risk*.”

“*Bridging the gap between uncertainty and helping to create an action plan for the producer*.”

We coded many more challenges related to producers than any other stakeholder group; we applied 20 of the 22 codes to this group (Table 2). Several of the coding themes related to complexities of farm management as compared with other groups (e.g., overworked, fear of regulation, risk management). Moreover, as noted above, challenges with timescale was mentioned by a few respondents described in relation to the producer stakeholder group.

“*Growers are focused on the immediate. They may recognize the need for longer term planning, but if they don’t make a crop this year, there will be no long term*.”

Many scientists also described challenges of communicating climate change issues as related to the way producers see the world (e.g., culture, politics). A few scientists wrote about implications of climate change to farm management (e.g., economics, fear of regulation, risk management).

“*…the realities of climate change go against a fundamental psychological requirement of being a farmer; always hoping and believing that next year will be better. Positively addressing climate change requires a positive attitude toward making changes in practices and expecting positive outcomes. Too much of the climate change message is negative because negative outcomes of climate change are what we talk about and it is difficult to be positive toward change if we like and are adjusted to what we are doing*.”

“*Crop producers do not want to change unless they are 100% sure the change will make money. That is almost impossible to do…*”

“*Many farmers/ranchers face such a large set of issues and complex decisions every single day that they barely have enough time to pause for lunch…they rarely have the luxury of enough spare time to develop a well-thought-out strategic (long-term) plan, let alone revisiting that plan regularly or incorporating new information or technology*.”

Finally, as reported in previous sections, many scientists indicated challenges in devising communication strategies for producers (e.g., making it personal, using layperson language). A few scientists also mentioned challenges surrounding how scientists can reach producers (e.g., pathway of information, pipeline to the right people, access to stakeholders).

“*A challenge is the ability to tell them what is the impact on them and their future family members by climate change that is and will happen in the future. Make it personal and not global. Also, talk about the changes that they have already made in relation to changing climate*.”

“*…I see the fundamental challenge is the limited number of opportunities for scientists and stakeholders to interact and share information…*”

## Discussion

Identifying strategies and challenges in scientists’ communication to agriculture stakeholder groups can aid in successful future communication [61]. Our respondents indicated experience working in a climate-related field with a diversity of agricultural stakeholders. We did not investigate how frequently they interacted with stakeholders, and how that would affect their responses. This would be important to investigate in further studies.

We did find that scientists are working with agricultural stakeholders whether they were trained to or not. The respondents also indicated scientists should be trained to effectively communicate scientific findings to nonscientists. This is consistent with other studies that suggest scientists should participate in communication training [34–36]. However, the belief (90.7%) and the practice (35.1%) are not congruent. Furthermore, training accessibility did not emerge as a challenge in the open response questions by stakeholder groups. This may indicate they do not find a lack of training, or accessibility to the training, as a challenge to communicating with the specific stakeholder or perhaps they do not believe the training is applicable to the stakeholder. Further research could investigate why training participation is not higher if it is valued. Are the barriers external (cost, not specific/applicable to stakeholder) to the participant or internal (time management/priority investment)? A limitation of the survey questionnaire is that respondents were not asked if they believed their communication strategies were successful. Despite the lack of training, does their experience supersede the value of training? Or perhaps, they believe that “other” scientists are in need of the training. A potential limitation of our survey is that respondents may be more invested in stakeholder engagement than non-respondents and our results to do not represent the breadth of scientist degree of communication and success of engagement.

Despite scientists having a different perspective than producers and crop advisors on almost all climate statements, they were able to estimate these stakeholders’ perspectives (Fig 1). We found scientists were able to predict most of the producers and all of the crop advisors’ level of agreement with the climate statements. Where there was a difference, it was not substantial. Note, that the surveyed scientists were a national audience and the producer and crop advisors were from 12 Midwestern states; however, our results are indicative scientists successfully understand their audiences’ perspective on climate and suggests this is not hindering communication between these groups. Additionally, the scientists’ perspective of other stakeholder groups such as agribusinesses or other geographical regions, should be explored to determine if a similar knowledge base exists. Additionally, due to the exploratory nature of this study, the statements were written as concise as possible to avoid participant survey fatigue. Future studies could parse out these climate change statements in greater detail to further explore the nuance in climate change perspectives and acceptance.

Scientists are deliberate with their choice of language when talking to different stakeholder groups. Different terminology use by term/phrase and target audience indicates a conscious decision to frame messages differently depending on target (Fig 2). Following best practices, scientists are making a deliberate effort to avoid jargon and consider the audience they are addressing to communicate more effectively; they specifically addressed terminology choice as a fundamental challenge to communicating climate change issues to most stakeholder groups. This strategy is consistent with others’ recommendations [33, 57, 61, 62, 67]. For example, phrases like “extreme weather” and “climate change” are more likely to be used than “global warming.” Additionally, “positive” and “negative” are not commonly used. Producers are a stakeholder group that scientists appear to be more cautious overall with terminology use, with seven of the eight significant terms/phrases less likely to be used with producers. This is consistent with previous research that found producers are generally considered to be a politically conservative group [80], and it is recommended to avoid controversial terms and issues (like climate change) with conservative audiences [8, 81–83]. Indeed, specific to communicating climate adaptive farm management strategies, some researchers recommend using terms like extreme weather and resilience rather than climate change risk [84, 85].

Morton, Roesch-McNally, and Wilke [71] found that 89.5% of producers in the Midwest could not justify changing their farm practices because they felt there was too much uncertainty about the impacts of climate change. Communicating about scientific uncertainty was identified as a common challenge with all stakeholder groups. Overall, however, farm management is a complicated endeavor, with a multitude of motivations and barriers that influence producers’ decision-making [86, 87]. Adding on concepts of climate change risk just adds to this complexity, and perhaps this is why others have recommended steering away from climate change as a good communication strategy. However, others have noted that ignoring realities of climate change risk by avoiding discussions specific to climate change may result in less urgency toward long-term strategies to increase agricultural resilience [19, 88, 89]. All of these challenges are in addition to the eroding of trust in science [51, 90, 91], which has been exacerbated by poor communication strategies during the COVID-19 pandemic [92], and the reality of how cultural and social norms relate to producers’ values that influence belief in climate science and their subsequent actions [53]. Although trust in science is eroding generally among both Democrats and Republicans in light of the COVID-19 pandemic, it is more dramatically eroding among Republicans [91]. This research has implications for producers’ trust in scientists, as producers across the U.S. tend to hold more conservative views [61].

Producers in the Midwestern U.S. seek information from specific sources; namely family members and agricultural advisors [93]. Distinct from scientists, agricultural advisors are the information intermediaries providing direct guidance to farmers on day-to-day farming decision-making by assessing farmers’ needs [68, 84, 94, 95]. Moreover, seeking and using information is positively related to producers adopting soil and water conservation practices [86] and trust in information sources is a motivation for conservation adoption [87]. These studies suggest the messenger, not only the message, is an important consideration for science communication. While Church et al. [19] found conservation and extension advisors were more likely to recommend adaptive management practices to their producers than financial and crop advisors, overall there is little evidence that agricultural advisors communicate agricultural climate change risk to their producers [19, 88]. There is a call for agricultural advisors and scientists (e.g., climatologists) to work together to build a shared understanding of agricultural climate change risk that can then be communicated to producers [19, 94, 96]. Our results suggest some promise in such a collaboration: scientists understand how to communicate climate risk with various stakeholders, as do agricultural advisors. Combining on-farm weather and climate information, and effective communication strategies could be a powerful catalyst for change.

The agricultural sector is particularly vulnerable to a changing climate and its effect can be seen from a local (disruption of planting schedules) to a global scale (food security). We found that scientists working in the agroclimate field are deliberate about their communication and are knowledgeable about their targeted stakeholders. This suggests methods for enhancing communication should be focused on other areas. Remaining challenges relate to already determined barriers to climate change communication including complexity [42, 43], uncertainty [62, 66, 67], and long-term climate change effects [33]. Our results show that though some challenges–stakeholder knowledge, timescale, and scientific uncertainty–are present in all of the stakeholder groups, others like developing a collective strategy or fear of regulation are very specific to one or two stakeholder groups. Specific to producers, we found scientists described challenges to communicating climate change issues as related to the fundamental job of farming/ranching. That is, producers are focused upon immediate decision-making and practices that allow them to manage their farms without disruption and ensure their livelihoods without incorporating new risks. These results correspond to other research which has found communicating short-term fixes to climate change related weather event impacts are preferred over pushing for long-term adaptive management strategies [19, 89]. These results also relate to the discussion above–changing farm practices due to perceived uncertainty in climate change risk is untenable [71].

Scientists in our study named stakeholder knowledge as a challenge for all stakeholder groups and suggested that education is the key to helping these stakeholder groups better understand climate science. This exemplifies how some scientists or messengers continue to use the knowledge deficit model to inform their communication methods and perspectives of public stakeholder groups [29], which may not be productive for moving agricultural stakeholders towards a consensus about the existence and causes of climate change. Although public understanding of science can play a role in acceptance of controversial types of science [97] and there are proven methods to enhance stakeholder knowledge of science (e.g., participatory adaptive management projects [98], using a community-based approach to managing science problems [99]), moving away from the knowledge deficit model of science communication could greatly enhance climate change communications between scientists and agricultural stakeholders. Encouraging climate scientists to attend communication trainings and practice communication methods grounded in social science research [29] could help climate scientists move away from using the knowledge deficit model to inform their communications with agricultural stakeholders. Co-production of knowledge could be another way of working toward adaptive management solutions, whereby producers, scientists, and agricultural advisors work on adaptive management strategies together to increase farm resilience and adapt to climate change [100–103].

Some of the difficulties of addressing climate change timescales with agricultural stakeholders could be dealt with via role-play simulations or serious games. In the past decade, many researchers have found role-play simulations and serious games can increase participant knowledge about climate change [104–107]. Addressing uncertainty in climate science is difficult, as there is the potential for stakeholders to misinterpret the usage of this word [66]. Some researchers suggest communicating transparently about what uncertainty means in the context of climate science, i.e., discussing the likelihoods of potential scenarios, the range of potential impacts, and the inherent uncertainty associated with all science can aid in preventing misinterpretations of uncertainty in the context of climate science [108]. Others suggest that in addition to this transparency, more cumbersome efforts of understanding stakeholders’ relevant decisions and how uncertainties in science can affect these decisions can aid in effective communication of uncertainty [63]. In addition to transparent communication, some research has shown simulations may be important tools in effective communication of uncertainty to non-science audiences [109].

## Supporting information

S1 File NIFA climate portfolio climate professional survey. (DOCX)

S1 Fig Scientist/professional type. Survey question was “please specify the type of scientist/professional you are (check all that apply):” Inset table indicates the frequency of respondents to select ≥1 value.
(DOCX)

S2 Fig Stakeholders currently or previously worked with. Survey question was “please select the following stakeholders that you currently or previously worked with (check all that apply).” Inset table indicates the frequency of respondents to select ≥1 value.
(DOCX)

S1 Table Scientist survey response rate. (DOCX)

S2 Table Scientists’ agreement with climate statements and their perspective on how stakeholders would respond. (DOCX)

S3 Table Scientists’ agreement with climate statements compared to producers and crop advisors’ agreement (Scientist perspectives 2017, Producer and Crop Advisor perspectives 2016^1^). (DOCX)

S4 Table Climate scientists’ perspective of the stakeholder compared to producers’ and crop advisors’ agreement on climate statements (Scientist perspectives 2017, Producer and Crop Advisor perspectives 2016^1^). (DOCX)

S5 Table Scientists’ likelihood of using terminology by stakeholder group sorted by overall decreasing likelihood ranking (mean). (DOCX)

S6 Table Communication challenges codes and examples (n = 227). (DOCX)
