# Supplementary material for: Understanding scientists’ communication challenges at the intersection of climate and agriculture
Source: PLoS One. 2022 Aug 2;17(8):e0269927. doi: 10.1371/journal.pone.0269927 (PMC9345487; doi:10.1371/journal.pone.0269927)
Supplement: S1 File — (DOCX) [file pone.0269927.s001.docx]

**NIFA Climate Portfolio Climate Professional Survey**

**NIFA Climate Change and Agroecosystems**

*Display: Introductory text and project Q1-Q7 only displayed to personnel identified through Project Direct Survey.*

You have been identified as a scientist who works on climate-related issues by a Project Director within the NIFA Climate Change and Agroecosystems Portfolio (Climate Portfolio).  The purpose of this survey is to gather your perspective on 1) Climate Portfolio projects, 2) the broad discipline of climate change and agroecosystems, and 3) climate change and agroecosystem stakeholders.  This synthesis will identify critical findings, lessons learned, and evaluate the effectiveness of the Climate Portfolio in promoting climate change and agroecosystem solutions.  Additionally, by participating in this survey you are assisting in identifying future funding priorities for the Climate Portfolio.

Your participation in this survey is voluntary.  We recognize that some of the questions may be sensitive; however, your answers will be kept confidential and will be released only as summaries where individual answers cannot be identified. The survey should take approximately 30-40 minutes to complete.  The system saves your progress as you go if you need to return at a later date to finish the survey.  Please read each question carefully.

For information regarding the survey, please contact Linda Prokopy (lprokopy@purdue.edu; 765-494-0825) or Jerry Hatfield (Jerry.Hatfield@ARS.USDA.GOV). Thank you in advance for your help!

**Introduction**

In this section, please respond to the following questions and confirm your status within the Climate Portfolio.

**project_question 1. (project_Q1).** **Are/were you involved with project account/accession number [ACCESSION NUMBER] entitled [PROPOSAL TITLE], a project within NIFA’s Climate Change and Agroecosystems Portfolio?**

- No
- Yes

*If respondent selected “No” responses not included in survey results..*

**project_Q2. Is this an on-going project?**

- No
- Yes

**project_Q3 *Have/Will* the following outcomes *been/be* achieved or not?**

|  | **Yes** | **No** |
| --- | --- | --- |
| Adaptation method(s)/strategy(-ies) to mitigate climate change impact on watersheds |  |  |
| Agricultural production method(s)/strategy(-ies) that can adapt to climate change |  |  |
| Agricultural science to optimize sustainable management of natural resources under a changing climate |  |  |
| Animal breed(s) that can adapt to climate change |  |  |
| Climate change impacts assessment data and/or method(s)/strategy(-ies) |  |  |
| Climate change impacts on water quality/quantity assessment data and/or method(s)/strategy(-ies) |  |  |
| Earth system model or component of an earth system model |  |  |
| Economic method(s)/strategy(-ies) to respond to climate change |  |  |
| Energy efficiency improvement method(s)/strategy(-ies) |  |  |
| Energy use reduction method(s)/strategy(-ies) |  |  |
| Extension and/or education method(s)/strategy(-ies) to communicate climate change issues to stakeholders |  |  |
| Forest management method(s)/strategy(-ies) for forestry to adapt to climate change |  |  |
| Forest production method(s)/strategy(-ies) that can adapt to climate change |  |  |
| Grazing land management method(s)/strategy(-ies) to adapt to climate change |  |  |
| Livestock production method(s)/strategy(-ies) that can adapt to climate change |  |  |
| Method(s)/strategy(-ies) to increase carbon sequestration |  |  |
| Method(s)/strategy(-ies) to increase the removal of CO_2_ and/or other greenhouse gasses from the atmosphere by natural processes |  |  |
| Natural resource conservation improvement method(s)/strategy(-ies) |  |  |
| New technology(-ies) |  |  |
| Nitrogen fertilizer loss reduction method(s)/strategy(-ies) |  |  |
| Nitrogen fertilizer use reduction method(s)/strategy(-ies) |  |  |
| Plant variety(-ies) that can adapt to climate change |  |  |
| Supply chain method(s)/strategy(-ies) for agriculture to adapt to climate change |  |  |
| Sustainable method(s)/strategy(-ies) that use natural resources |  |  |

**project_Q4** **If not listed above, what outcomes *have been/will be* developed through this project?**

**project_Q5** **In your opinion, what is the largest contribution of your project relative to climate change and agroecosystems?**

**project_Q6 Based on your project findings, what do you believe to be the remaining knowledge gaps?**

**project_Q7 Please use the space below for any additional comments regarding this project.**

**The remainder of this survey does not pertain to your project within the Climate Portfolio.  Once you click the next arrow (>>), you will proceed and not be able to return to this project-specific section.  The remainder of this survey pertains to climate and agroecosystem work in general.**

*Display: Introductory text if personnel not identified through Project Direct Survey.*

**NIFA Climate Change and Agroecosystems Synthesis**

You have been identified as a scientist who works on climate-related issues due to your role as ${e://Field/title_role} within the ${e://Field/organization}.  The purpose of this survey is to gather your perspective on 1) the broad discipline of climate change and agroecosystems and 2) climate change and agroecosystem stakeholders.  This synthesis will assist in the evaluation of the effectiveness of the NIFA Climate Change and Agroecosystems Portfolio (Climate Portfolio) in promoting climate change and agroecosystem solutions.  Additionally, by participating in this survey you are assisting in identifying future funding priorities for the Climate Portfolio.

Your participation in this survey is voluntary.  We recognize that some of the questions may be sensitive; however, your answers will be kept confidential and will be released only as summaries where individual answers cannot be identified. The survey should take approximately 30-40 minutes to complete.  The system saves your progress as you go if you need to return at a later date to finish the survey.  Please read each question carefully.

For information regarding the survey, please contact Linda Prokopy (lprokopy@purdue.edu; 765-494-0825) or Jerry Hatfield (Jerry.Hatfield@ARS.USDA.GOV). Thank you in advance for your help!

**Section 1: Climate Change and Agroecosystems**

**We seek to understand the broader discipline of climate change and agroecosystems.  Please answer the following questions regarding the field of climate change and agroecosystems science.**

**climate_change_Q1 Please indicate your level of agreement with the following statements.**

|  | **Strongly disagree** | **Disagree** | **Neither agree nor disagree** | **Agree** | **Strongly agree** |
| --- | --- | --- | --- | --- | --- |
| Earth’s climate conditions occur at random with no cycles or trends. |  |  |  |  |  |
| Earth’s climate conditions occur in a cyclical pattern. |  |  |  |  |  |
| Even if climate changes, we cannot predict what those changes will be in the future. |  |  |  |  |  |
| Climate change is happening. |  |  |  |  |  |
| Earth’s climate always changes. |  |  |  |  |  |
| Human activities are contributing to climate change. |  |  |  |  |  |
| Human activities are the primary driver of climate change. |  |  |  |  |  |
| Climate change will not affect the way that I live. |  |  |  |  |  |
| There is enough evidence that climate is changing. |  |  |  |  |  |
| The majority of the public distrusts scientists that work on climate-related issues. |  |  |  |  |  |

**climate_change_Q2 In your opinion, what are up to 5 key areas to focus climate and agroecosystem research in the next decade?**

**climate_change_Q3 In your opinion, what is the ideal spatial scale to research the key areas you described above?**

**climate_change_Q4 In your opinion, how long (in years) should a funding cycle be to research climate change (please enter a numeric value)?**

**climate_change_Q5 Please indicate your level of agreement with the following statements in terms of climate change and agroecosystem science.**

|  | **Strongly disagree** | **Disagree** | **Neither agree nor disagree** | **Agree** | **Strongly agree** |
| --- | --- | --- | --- | --- | --- |
| Climate change mitigation and/or adaptation strategies are on track and desired outcomes will be forthcoming. |  |  |  |  |  |
| Climate change mitigation and/or adaptation strategies should focus on maximizing resilience rather than relying on climate model projections. |  |  |  |  |  |
| Climate change policy should focus on promoting collective action. |  |  |  |  |  |
| Climate model projections reflect too large of a scale to be useful to stakeholders. |  |  |  |  |  |
| Climate model projections should focus on refining model accuracy to reduce the range of model outputs. |  |  |  |  |  |
| Climate model projections should not be used to inform mitigation and/or adaptation strategies. |  |  |  |  |  |
| Disinformation campaigns contribute to the lack of climate change mitigation and/or adaptation strategy implementation. |  |  |  |  |  |
| Media support is necessary for development of climate change mitigation and/or adaptation strategies. |  |  |  |  |  |
| Media support is necessary for implementation of climate change mitigation and/or adaptation strategies. |  |  |  |  |  |
| Policymakers need comprehensible scientific input to inform their decision making. |  |  |  |  |  |
| Policymakers should frame climate change policy on the long-term consequences of inaction. |  |  |  |  |  |
| Policymakers should frame climate change policy on the short-term benefits of immediate action. |  |  |  |  |  |
| Political support is necessary for development of climate change mitigation and/or adaptation strategies. |  |  |  |  |  |
| Political support is necessary for implementation of climate change mitigation and/or adaptation strategies. |  |  |  |  |  |
| Public scientific illiteracy contributes to the lack of climate change mitigation and/or adaptation strategy implementation. |  |  |  |  |  |
| Public support is necessary for development of climate change mitigation and/or adaptation strategies. |  |  |  |  |  |
| Public support is necessary for implementation of climate change mitigation and/or adaptation strategies. |  |  |  |  |  |

**climate_change_Q6 Please indicate your level of agreement with the following statements.**

|  | **Strongly disagree** | **Disagree** | **Neither agree nor disagree** | **Agree** | **Strongly agree** |
| --- | --- | --- | --- | --- | --- |
| Scientists have a responsibility to society to provide scientific input to policymakers. |  |  |  |  |  |
| Scientists have a responsibility to society to provide scientific input to the public. |  |  |  |  |  |
| Scientists should advocate for climate change mitigation and/or adaptation policy. |  |  |  |  |  |
| Scientists should conduct agricultural stakeholder needs assessments. |  |  |  |  |  |
| Scientists should contribute to climate change mitigation and/or adaptation policy. |  |  |  |  |  |
| Scientists should develop/create climate change mitigation and/or adaptation policy. |  |  |  |  |  |
| Scientists should distinguish between uncertainty and variability when communicating with nonscientists. |  |  |  |  |  |
| Scientists should drive climate change mitigation and/or adaptation solutions. |  |  |  |  |  |
| Scientists should never refer to known variation as uncertainty. |  |  |  |  |  |
| Scientists should receive training on how to communicate scientific findings to nonscientists. |  |  |  |  |  |

**Section 2: Stakeholders**

**We seek to understand how information is disseminated to the following stakeholders. Stakeholders are persons or groups that have an interest or concern in your work topic(s), finding(s), and/or outcome(s).  Please answer the following questions regarding stakeholders.**

**stakeholder_Q1 Have you received formal training on how to communicate with stakeholders?**

- No
- Yes

*Display: If respondent selected “Yes” to stakeholders_Q1, stakeholders_Q2 would display.*

**stakeholder_Q2 Please describe the *formal* training you received on communicating with stakeholders.**

**stakeholder_Q3 Please describe any *informal* training and/or research you have done to improve your ability to communicate with stakeholders.**

**stakeholder_Q4 Please select the following stakeholders that you currently or previously worked with (check all that apply).  If none, click the next arrow (>>) to skip.**

- Agribusinesses
- Crop advisors
- Crop and/or livestock producers
- General public
- Policymakers

**In the following section you will receive a series of questions that correspond to the stakeholder(s) you selected in the previous section.**

*Display: If respondent selected more than one stakeholder in stakeholder_Q1, stakeholder_Q4 would display.*

**stakeholder_Q5 In the previous section you indicated that you worked with multiple stakeholders, in an attempt to shorten this survey we will ask you a series of questions regarding the stakeholder that is your main priority, which stakeholder is your main priority?**

- Agribusinesses
- Crop advisors
- Crop and/or livestock producers
- General public
- Policymakers

*Display: If respondent selected agribusiness in stakeholder_Q4 as single response, stakeholder_Q5 as the primary stakeholder, or in multi-stakeholder_2, display agribusiness questions 1-5.*

**Agribusinesses**

**agribusiness_Q1 How far (in years) would you model climate projections to maximize usefulness to *agribusinesses* (please enter a numeric value)?**

**agribusiness_Q2 In your opinion, what is the fundamental challenge in communicating climate change issues to *agribusinesses*?**

**agribusiness_Q3 How likely are you to use the following terms/phrases when talking to *agribusinesses* about climate change?**

|  | **Extremely unlikely** | **Unlikely** | **Neither likely nor unlikely** | **Likely** | **Extremely likely** |
| --- | --- | --- | --- | --- | --- |
| Climate change |  |  |  |  |  |
| Climate debate |  |  |  |  |  |
| Climate variability |  |  |  |  |  |
| Error |  |  |  |  |  |
| Extreme weather |  |  |  |  |  |
| Global warming |  |  |  |  |  |
| Green development |  |  |  |  |  |
| Greenhouse gases |  |  |  |  |  |
| Heterogeneity |  |  |  |  |  |
| Holistic approach |  |  |  |  |  |
| Long-term weather |  |  |  |  |  |
| Manmade and/or human made |  |  |  |  |  |
| Negative trend |  |  |  |  |  |
| Positive trend |  |  |  |  |  |
| Rapid change |  |  |  |  |  |
| Resiliency |  |  |  |  |  |
| Social-ecological systems |  |  |  |  |  |
| Stress |  |  |  |  |  |
| Sustainability |  |  |  |  |  |
| Theory |  |  |  |  |  |
| Uncertainty |  |  |  |  |  |
| Unusual weather |  |  |  |  |  |
| Variability |  |  |  |  |  |
| Weather variability |  |  |  |  |  |

**agribusiness_Q4 Are there terms/phrases that you *avoid* using when talking to *agribusinesses* about climate change?**

**agribusiness_Q5 In your opinion, what would the majority of *agribusinesses'* level of agreement be with the following statements?**

|  | **Strongly disagree** | **Disagree** | **Neither agree nor disagree** | **Agree** | **Strongly agree** |
| --- | --- | --- | --- | --- | --- |
| Earth’s climate conditions occur at random with no cycles or trends. |  |  |  |  |  |
| Earth’s climate conditions occur in a cyclical pattern. |  |  |  |  |  |
| Even if climate changes, we cannot predict what those changes will be in the future. |  |  |  |  |  |
| Climate change is happening. |  |  |  |  |  |
| Earth’s climate always changes. |  |  |  |  |  |
| Human activities are contributing to climate change. |  |  |  |  |  |
| Human activities are the primary driver of climate change. |  |  |  |  |  |
| Climate change will not affect the way that agribusinesses operate. |  |  |  |  |  |
| There is enough evidence that climate is changing. |  |  |  |  |  |
| Agribusinesses distrust scientists that work on climate-related issues. |  |  |  |  |  |

*Display: If respondent selected crop advisors in stakeholder_Q4 as single response, stakeholder_Q5 as the primary stakeholder, or in multi-stakeholder_2 , display crop advisor questions 1-5.*

**Crop Advisors**

**crop_advisor_Q1 How far (in years) would you model climate projections to maximize usefulness to *crop advisors* (please enter a numeric value)?**

**crop_advisor_Q2 In your opinion, what is the fundamental challenge in communicating climate change issues to *crop advisors*?**

**crop_advisor_Q3 How likely are you to use the following terms/phrases when talking to *crop advisors* about climate change?**

|  | **Extremely unlikely** | **Unlikely** | **Neither likely nor unlikely** | **Likely** | **Extremely likely** |
| --- | --- | --- | --- | --- | --- |
| Climate change |  |  |  |  |  |
| Climate debate |  |  |  |  |  |
| Climate variability |  |  |  |  |  |
| Error |  |  |  |  |  |
| Extreme weather |  |  |  |  |  |
| Global warming |  |  |  |  |  |
| Green development |  |  |  |  |  |
| Greenhouse gases |  |  |  |  |  |
| Heterogeneity |  |  |  |  |  |
| Holistic approach |  |  |  |  |  |
| Long-term weather |  |  |  |  |  |
| Manmade and/or human made |  |  |  |  |  |
| Negative trend |  |  |  |  |  |
| Positive trend |  |  |  |  |  |
| Rapid change |  |  |  |  |  |
| Resiliency |  |  |  |  |  |
| Social-ecological systems |  |  |  |  |  |
| Stress |  |  |  |  |  |
| Sustainability |  |  |  |  |  |
| Theory |  |  |  |  |  |
| Uncertainty |  |  |  |  |  |
| Unusual weather |  |  |  |  |  |
| Variability |  |  |  |  |  |
| Weather variability |  |  |  |  |  |

**crop_advisor_Q4** **Are there terms/phrases that you *avoid* using when talking to *crop advisors* about climate change?**

**crop_advisors_Q5** **In your opinion, what would the majority of *crop advisors'* level of agreement be with the following statements?**

|  | **Strongly disagree** | **Disagree** | **Neither agree nor disagree** | **Agree** | **Strongly agree** |
| --- | --- | --- | --- | --- | --- |
| Earth’s climate conditions occur at random with no cycles or trends. |  |  |  |  |  |
| Earth’s climate conditions occur in a cyclical pattern. |  |  |  |  |  |
| Even if climate changes, we cannot predict what those changes will be in the future. |  |  |  |  |  |
| Climate change is happening. |  |  |  |  |  |
| Earth’s climate always changes. |  |  |  |  |  |
| Human activities are contributing to climate change. |  |  |  |  |  |
| Human activities are the primary driver of climate change. |  |  |  |  |  |
| Climate change will not affect the way that crop advisors operate. |  |  |  |  |  |
| There is enough evidence that climate is changing. |  |  |  |  |  |
| Crop advisors distrust scientists that work on climate-related issues. |  |  |  |  |  |

*Display: If respondent selected producers in stakeholder_Q4 as single response, stakeholder_Q5 as the primary stakeholder, or in multi-stakeholder_2, display producer questions 1-5.*

**Producers**

**producers_Q1 How far (in years) would you model climate projections to maximize usefulness to *crop and/or livestock producers* (please enter a numeric value)?**

**producers_Q2 In your opinion, what is the fundamental challenge in communicating climate change issues to *crop and/or livestock producers*?**

**producers_Q3 How likely are you to use the following terms/phrases when talking to *crop and/or livestock producers* about climate change?**

|  | **Extremely unlikely** | **Unlikely** | **Neither likely nor unlikely** | **Likely** | **Extremely likely** |
| --- | --- | --- | --- | --- | --- |
| Climate change |  |  |  |  |  |
| Climate debate |  |  |  |  |  |
| Climate variability |  |  |  |  |  |
| Error |  |  |  |  |  |
| Extreme weather |  |  |  |  |  |
| Global warming |  |  |  |  |  |
| Green development |  |  |  |  |  |
| Greenhouse gases |  |  |  |  |  |
| Heterogeneity |  |  |  |  |  |
| Holistic approach |  |  |  |  |  |
| Long-term weather |  |  |  |  |  |
| Manmade and/or human made |  |  |  |  |  |
| Negative trend |  |  |  |  |  |
| Positive trend |  |  |  |  |  |
| Rapid change |  |  |  |  |  |
| Resiliency |  |  |  |  |  |
| Social-ecological systems |  |  |  |  |  |
| Stress |  |  |  |  |  |
| Sustainability |  |  |  |  |  |
| Theory |  |  |  |  |  |
| Uncertainty |  |  |  |  |  |
| Unusual weather |  |  |  |  |  |
| Variability |  |  |  |  |  |
| Weather variability |  |  |  |  |  |

**producers_Q4 Are there terms/phrases that you *avoid* using when talking to *crop and/or livestock producers* about climate change?**

**producers_Q5 In your opinion, what would the majority of *crop and/or livestock producers'*level of agreement be with the following statements?**

|  | **Strongly disagree** | **Disagree** | **Neither agree nor disagree** | **Agree** | **Strongly agree** |
| --- | --- | --- | --- | --- | --- |
| Earth’s climate conditions occur at random with no cycles or trends. |  |  |  |  |  |
| Earth’s climate conditions occur in a cyclical pattern. |  |  |  |  |  |
| Even if climate changes, we cannot predict what those changes will be in the future. |  |  |  |  |  |
| Climate change is happening. |  |  |  |  |  |
| Earth’s climate always changes. |  |  |  |  |  |
| Human activities are contributing to climate change. |  |  |  |  |  |
| Human activities are the primary driver of climate change. |  |  |  |  |  |
| Climate change will not affect the way that crop and/or livestock producers operate. |  |  |  |  |  |
| There is enough evidence that climate is changing |  |  |  |  |  |
| Crop and/or livestock producers distrust scientists that work on climate-related issues. |  |  |  |  |  |

*Display: If respondent selected general public in stakeholder_Q4 as single response, stakeholder_Q5 as the primary stakeholder, or in multi-stakeholder_2, display general public questions 1-5.*

**General Public**

**public_Q1 How far (in years) would you model climate projections to maximize usefulness to the *general public* (please enter a numeric value)?**

**public_Q2 In your opinion, what is the fundamental challenge in communicating climate change issues to the general public?**

**public_Q3 How likely are you to use the following terms/phrases when talking to the general public about climate change?**

|  | **Extremely unlikely** | **Unlikely** | **Neither likely nor unlikely** | **Likely** | **Extremely likely** |
| --- | --- | --- | --- | --- | --- |
| Climate change |  |  |  |  |  |
| Climate debate |  |  |  |  |  |
| Climate variability |  |  |  |  |  |
| Error |  |  |  |  |  |
| Extreme weather |  |  |  |  |  |
| Global warming |  |  |  |  |  |
| Green development |  |  |  |  |  |
| Greenhouse gases |  |  |  |  |  |
| Heterogeneity |  |  |  |  |  |
| Holistic approach |  |  |  |  |  |
| Long-term weather |  |  |  |  |  |
| Manmade and/or human made |  |  |  |  |  |
| Negative trend |  |  |  |  |  |
| Positive trend |  |  |  |  |  |
| Rapid change |  |  |  |  |  |
| Resiliency |  |  |  |  |  |
| Social-ecological systems |  |  |  |  |  |
| Stress |  |  |  |  |  |
| Sustainability |  |  |  |  |  |
| Theory |  |  |  |  |  |
| Uncertainty |  |  |  |  |  |
| Unusual weather |  |  |  |  |  |
| Variability |  |  |  |  |  |
| Weather variability |  |  |  |  |  |

**public_Q4 Are there terms/phrases that you *avoid* using when talking to the *general public* about climate change?**

**public_Q5 In your opinion, what would the majority of the *general public's*level of agreement be with the following statements?**

|  | **Strongly disagree** | **Disagree** | **Neither agree nor disagree** | **Agree** | **Strongly agree** |
| --- | --- | --- | --- | --- | --- |
| Earth’s climate conditions occur at random with no cycles or trends. |  |  |  |  |  |
| Earth’s climate conditions occur in a cyclical pattern. |  |  |  |  |  |
| Even if climate changes, we cannot predict what those changes will be in the future. |  |  |  |  |  |
| Climate change is happening. |  |  |  |  |  |
| Earth’s climate always changes. |  |  |  |  |  |
| Human activities are contributing to climate change. |  |  |  |  |  |
| Human activities are the primary driver of climate change. |  |  |  |  |  |
| Climate change will not affect the way that the general public lives. |  |  |  |  |  |
| There is enough evidence that climate is changing. |  |  |  |  |  |
| The general public distrusts scientists that work on climate-related issues. |  |  |  |  |  |

*Display: If respondent selected policymakers in stakeholder_Q4 as single response, stakeholder_Q5 as the primary stakeholder, or in multi-stakeholder_2, display policymaker questions 1-5.*

**Policymakers**

**policymaker_Q1 How far (in years) would you model climate projections to maximize usefulness to *policymakers* (please enter a numeric value)?**

**policymaker_Q2 In your opinion, what is the fundamental challenge in communicating climate change issues to *policymakers*?**

**policymaker_Q3 How likely are you to use the following terms/phrases when talking to *policymakers*about climate change?**

|  | **Extremely unlikely** | **Unlikely** | **Neither likely nor unlikely** | **Likely** | **Extremely likely** |
| --- | --- | --- | --- | --- | --- |
| Climate change |  |  |  |  |  |
| Climate debate |  |  |  |  |  |
| Climate variability |  |  |  |  |  |
| Error |  |  |  |  |  |
| Extreme weather |  |  |  |  |  |
| Global warming |  |  |  |  |  |
| Green development |  |  |  |  |  |
| Greenhouse gases |  |  |  |  |  |
| Heterogeneity |  |  |  |  |  |
| Holistic approach |  |  |  |  |  |
| Long-term weather |  |  |  |  |  |
| Manmade and/or human made |  |  |  |  |  |
| Negative trend |  |  |  |  |  |
| Positive trend |  |  |  |  |  |
| Rapid change |  |  |  |  |  |
| Resiliency |  |  |  |  |  |
| Social-ecological systems |  |  |  |  |  |
| Stress |  |  |  |  |  |
| Sustainability |  |  |  |  |  |
| Theory |  |  |  |  |  |
| Uncertainty |  |  |  |  |  |
| Unusual weather |  |  |  |  |  |
| Variability |  |  |  |  |  |
| Weather variability |  |  |  |  |  |

**policymaker_Q4 Are there terms/phrases that you *avoid* using when talking to *policymakers*about climate change?**

**policymaker_Q5 In your opinion, what would the majority of *policymakers'* level of agreement be with the following statements?**

|  | **Strongly disagree** | **Disagree** | **Neither agree nor disagree** | **Agree** | **Strongly agree** |
| --- | --- | --- | --- | --- | --- |
| Earth’s climate conditions occur at random with no cycles or trends. |  |  |  |  |  |
| Earth’s climate conditions occur in a cyclical pattern. |  |  |  |  |  |
| Even if climate changes, we cannot predict what those changes will be in the future. |  |  |  |  |  |
| Climate change is happening. |  |  |  |  |  |
| Earth’s climate always changes. |  |  |  |  |  |
| Human activities are contributing to climate change. |  |  |  |  |  |
| Human activities are the primary driver of climate change. |  |  |  |  |  |
| Climate change will not affect the way that policymakers operate. |  |  |  |  |  |
| There is enough evidence that climate is changing. |  |  |  |  |  |
| Policymakers distrust scientists that work on climate-related issues. |  |  |  |  |  |

**Multi-stakeholder**

*Display: If respondent selected multiple stakeholders in stakeholder_Q4, display multi-stakeholder_Q1.*

**multi-stakeholder_1 You indicated that you worked with multiple stakeholder types. You are welcome to answer the same block of questions about communicating to other stakeholder groups. Please be aware that the remainder of this survey is a brief demographic section.**

- No, I am not interested in answering additional stakeholder questions.
- Yes, I am interested in answering the five stakeholder questions for additional group(s).

*Display: If respondent selected “*Yes, I am interested in answering the five stakeholder questions for additional group(s).” *in stakeholder_Q1, display multi-stakeholder_Q2.*

**multi-stakeholder_Q2** **Please select the stakeholder group(s) (check all that apply).  If more than one group is displayed below, please select all the stakeholder groups that you are interested in answering the stakeholder questions for.**

- Agribusinesses
- Crop advisors
- Crop and/or livestock producers
- General public
- Policymakers

**Section 3: Demographics**

**demographic_Q1 What year were you born?**

**demographic _Q2 What is your gender?**

*Display: If respondent was identified through Project Direct Survey, display demographic_Q3.*

**demographic_Q3 What is your job title (check all that apply)?**

- Assistant Professor
- Associate Professor
- Chair
- Dean
- Extension Specialist/Educator
- Full Professor
- Postdoctoral Associate
- Program Director/Manager
- Research Associate/Assistant Professor
- Research Staff (i.e. Scientist, Specialist, Technician, etc.)
- Other (please specify): ________________________________________________

**demographic_Q4 Please specify the type of scientist/professional you are (check all that apply):**

- Climate scientist
- Computer scientist
- Economist
- Educator
- Engineer
- Extension specialist/educator
- Geospatial scientist
- Legal scholar/professional
- Life scientist (e.g. biologist, ecologist, botanist, zoologist, physiologist, biochemist or related subject) (please specify): ________________________________________________
- Mathematician/Statistician
- Physical scientist (excluding climate scientist) (e.g. chemist, astronomer, geologist, physicist or related subject) (please specify): ________________________________________________
- Public Health scientist/professional
- Social scientist (non-economist)
- Other (please specify): ________________________________________________

**demographic_Q5 Please indicate how many years you have been working in a climate-related field as your occupation (please enter a numeric value):**

**demographic_Q6 Please indicate the approximate percentage of time you spend on climate-related work as part of your job (please enter a numeric value):**

**demographic_Q7 Please indicate the number of years you have been working in your current role (please enter a numeric value):**

*Display: If respondent was not identified through Project Direct Survey and not a State Climatologist, display demographic_Q8.*

**demographic_Q8 Do you have a role in making climate-related funding decisions in your current agency position?**

- No
- Yes

*Display: If respondent selected “Yes” in* *demographic_Q8, display demographic_Q9.*

**demographic_Q9 What is your current decision making role for climate-related funding?**

**demographic_Q10 Do you have additional comments about this survey, climate change and agroecosystem science, and/or stakeholders?**

**Thank you for completing this survey and providing your perspectives on climate change and agroecosystems. Once you click the next arrow (>>) your responses will be submitted.  We may contact you in the future for additional input and information.  For information regarding the survey, please contact Linda Prokopy (lprokopy@purdue.edu; 765-494-0825) or Jerry Hatfield (Jerry.Hatfield@ARS.USDA.GOV).**
