# Supplementary material for: Understanding scientists’ communication challenges at the intersection of climate and agriculture
Source: PLoS One. 2022 Aug 2;17(8):e0269927. doi: 10.1371/journal.pone.0269927 (PMC9345487; doi:10.1371/journal.pone.0269927)
Supplement: S1 Fig — Survey question was “please specify the type of scientist/professional you are (check all that apply):” Inset table indicates the frequency of respondents to select ≥1 value. (DOCX) [file pone.0269927.s002.docx]

**
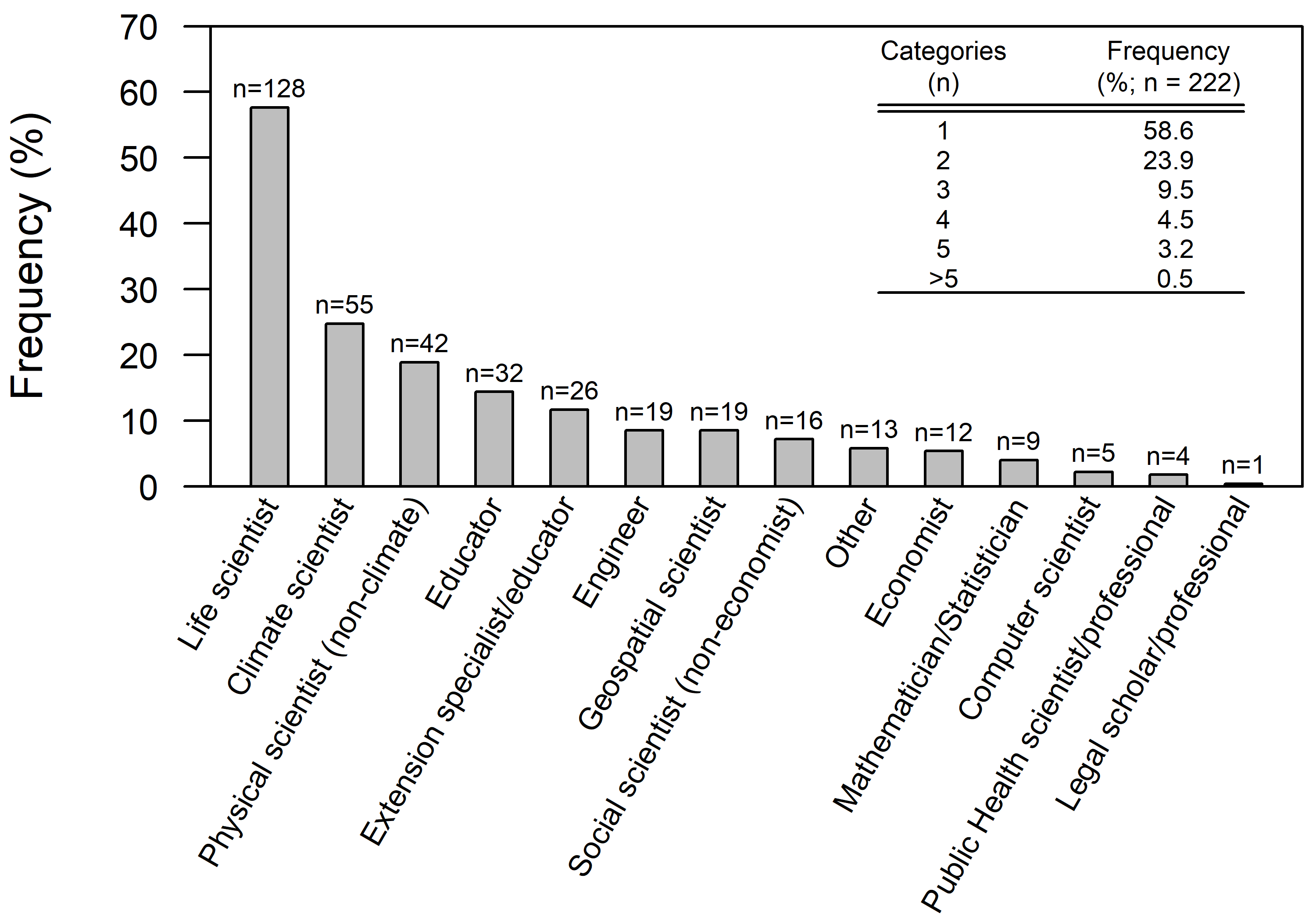
**

**Fig 1. Scientist/professional type.** Survey question was “please specify the type of scientist/professional you are (check all that apply):” Inset table indicates the frequency of respondents to select ≥1 value.
