# Supplementary material for: Understanding scientists’ communication challenges at the intersection of climate and agriculture
Source: PLoS One. 2022 Aug 2;17(8):e0269927. doi: 10.1371/journal.pone.0269927 (PMC9345487; doi:10.1371/journal.pone.0269927)
Supplement: S1 Table — (DOCX) [file pone.0269927.s004.docx]

| **Table 1. Scientist Survey Response Rate** | | | |
| --- | --- | --- | --- |
| **Recipient group** | **Completed (n)** | **Eligible (n)** | **Response Rate (%)** |
| Government/AASC | 105 | 331 | 31.7 |
| NIFA Portfolio | 160 | 288 | 55.6 |
| **Total** | **265** | **619** | **42.8** |
