# Supplementary material for: Understanding scientists’ communication challenges at the intersection of climate and agriculture
Source: PLoS One. 2022 Aug 2;17(8):e0269927. doi: 10.1371/journal.pone.0269927 (PMC9345487; doi:10.1371/journal.pone.0269927)
Supplement: S2 Table — (DOCX) [file pone.0269927.s005.docx]

**Table 2. Scientists’ agreement with climate statements and their perspective on how stakeholders would respond**

| **Statement** | **Scientists** | | **Agribusinesses** | | | **Crop Advisors** | | | **Producers** | | | **Public** | | | **Policymakers** | | | **p-value** |
| --- | --- | --- | --- | --- | --- | --- | --- | --- | --- | --- | --- | --- | --- | --- | --- | --- | --- | --- |
|  | ***n*** | ***M***  ***Mdn*** | ***n*** | ***M***  ***Mdn*** | ***p*** | ***n*** | ***M***  ***Mdn*** | ***p*** | ***n*** | ***M***  ***Mdn*** | ***p*** | ***n*** | ***M***  ***Mdn*** | ***p*** | ***N*** | ***M***  ***Mdn*** | ***p*** |  |
| Earth's climate conditions occur at random with no cycles or trends. | 245 | 1.4 | 20 | 2.4 | *** | 10 | 2.3 | * | 72 | 2.7 | *** | 69 | 2.8 | *** | 57 | 2.5 | *** | <0.001 |
|  |  | 1 |  | 2 |  |  | 2 |  |  | 3 |  |  | 3 |  |  | 2 |  |  |
| Earth's climate conditions occur in a cyclical pattern. | 243 | 3.5 | 20 | 3.6 |  | 10 | 3.8 |  | 71 | 3.6 |  | 69 | 3.5 |  | 57 | 3.6 |  | 0.626 |
|  |  | 4 |  | 4 |  |  | 4 |  |  | 4 |  |  | 4 |  |  | 4 |  |  |
| Even if climate changes, we cannot predict what those changes will be in the future. | 243 | 2.1 | 20 | 3.4 | *** | 10 | 2.9 | * | 72 | 3.4 | *** | 69 | 3.2 | *** | 57 | 3.1 | *** | <0.001 |
|  |  | 2 |  | 3 |  |  | 3 |  |  | 4 |  |  | 3 |  |  | 3 |  |  |
| Climate change is happening. | 242 | 4.7 | 20 | 4 | *** | 10 | 3.8 | *** | 72 | 3.6 | *** | 69 | 3.9 | *** | 56 | 3.7 | *** | <0.001 |
|  |  | 5 |  | 4 |  |  | 4 |  |  | 4 |  |  | 4 |  |  | 4 |  |  |
| Earth's climate always changes. | 242 | 4.1 | 20 | 4 |  | 10 | 4.1 |  | 72 | 3.9 |  | 69 | 3.9 | * | 57 | 4 |  | 0.051 |
|  |  | 4 |  | 4 |  |  | 4 |  |  | 4 |  |  | 4 |  |  | 4 |  |  |
| Human activities are contributing to climate change. | 242 | 4.7 | 20 | 3.5 | *** | 10 | 3.6 | *** | 72 | 3.2 | *** | 69 | 3.7 | *** | 57 | 3.5 | *** | <0.001 |
|  |  | 5 |  | 4 |  |  | 3.5 |  |  | 3 |  |  | 4 |  |  | 4 |  |  |
| Human activities are the primary driver of climate change. | 242 | 4.2 | 20 | 3 | *** | 10 | 3.1 | * | 72 | 2.6 | *** | 69 | 3.3 | *** | 57 | 2.9 | *** | <0.001 |
|  |  | 4 |  | 3 |  |  | 3 |  |  | 3 |  |  | 3 |  |  | 3 |  |  |
| Climate change will not affect the way that the [stakeholders] [operate/lives]. | 242 | 1.7 | 20 | 2 |  | 10 | 2.3 |  | 72 | 2.3 | *** | 69 | 3 | *** | 57 | 2.9 | *** | <0.001 |
|  |  | 1.5 |  | 2 |  |  | 2 |  |  | 2 |  |  | 3 |  |  | 3 |  |  |
| There is enough evidence that climate is changing. | 242 | 4.4 | 20 | 3.6 | *** | 10 | 3.5 | ** | 72 | 3.2 | *** | 69 | 3.4 | *** | 57 | 3.3 | *** | <0.001 |
|  |  | 5 |  | 4 |  |  | 3.5 |  |  | 3 |  |  | 4 |  |  | 3 |  |  |
| [Stakeholder] distrust scientists that work on climate-related issues. | 242 | 2.5 | 20 | 2.8 |  | 10 | 2.6 |  | 72 | 3.1 | *** | 69 | 2.8 |  | 57 | 2.9 | * | <0.001 |
|  |  | 2 |  | 3 |  |  | 2.5 |  |  | 3 |  |  | 3 |  |  | 3 |  |  |
| Notes. Survey questions: “Please indicate your level of agreement with the following statements” and “In your opinion, what would the majority of [stakeholder’s] level of agreement be with the following statements?” Two statements were rephrased to be specific to each stakeholder (indicated with brackets). All stakeholder group statements displayed “operate” except the general public. The statements that asked about the scientist’s own perspective were “Climate change will not affect the way that I live” and “The majority of the public distrusts scientists that work on climate related issues.”  scale: 1 = strongly disagree, 2 = disagree, 3 = neither agree nor disagree, 4 = agree, 5 = strongly agree  "p" column (Wilcoxon-Mann-Whitney test [two-sided] adjusted by Bonferroni correction method for multiple comparison) indicates the statistical significance of the climate scientists’ agreement with each climate statement comparing the climate scientists’ perspective of how the stakeholders would respond to the same climate statement; *, **, and *** corresponds to a statistical significance level at 0.05, 0.01, and 0.001 respectively. The p-value column (Kruskal-Wallis test) indicates whether the distribution of the responses for all six groups were different overall by statement. | | | | | | | | | | | | | | | | | | |
