# Supplementary material for: Understanding scientists’ communication challenges at the intersection of climate and agriculture
Source: PLoS One. 2022 Aug 2;17(8):e0269927. doi: 10.1371/journal.pone.0269927 (PMC9345487; doi:10.1371/journal.pone.0269927)
Supplement: S3 Table — (DOCX) [file pone.0269927.s006.docx]

**Table 3. Scientists’ agreement with climate statements compared to producers and crop advisors’ agreement (Scientist perspectives 2017, Producer and Crop Advisor perspectives 2016^1^)**

| **Statement** | **Scientists** | | | **Producers^1^** | | | **Crop Advisors^1^** | | | **p-value** |
| --- | --- | --- | --- | --- | --- | --- | --- | --- | --- | --- |
|  | ***n*** | ***M***  ***Mdn*** | ***p*** | ***n*** | ***M***  ***Mdn*** | ***p*** | ***n*** | ***M***  ***Mdn*** | ***p*** |  |
| Earth's climate conditions occur at random with no cycles or trends. | 245 | 1.4 | ***a ***b | 1388 | 2.6 | ***a ***c | 1969 | 2.2 | ***b ***c | <0.001 |
|  |  | 1 |  |  | 2 |  |  | 2 |  |  |
| Earth's climate conditions occur in a cyclical pattern. | 243 | 3.5 |  | 1383 | 3.6 | ***a | 1964 | 3.7 | ***a | <0.001 |
|  |  | 4 |  |  | 4 |  |  | 4 |  |  |
| Even if climate changes, we cannot predict what those changes will be in the future. | 243 | 2.1 | ***a ***b | 1383 | 3.5 | ***a ***c | 1962 | 3.1 | ***b ***c | <0.001 |
|  |  | 2 |  |  | 4 |  |  | 3 |  |  |
| Climate change is happening. | 242 | 4.7 | ***a ***b | 1383 | 3.4 | ***a ***c | 1966 | 3.7 | ***b ***c | <0.001 |
|  |  | 5 |  |  | 4 |  |  | 4 |  |  |
| Earth's climate always changes. | 242 | 4.1 | ***a  *b | 1384 | 4 | ***a *c | 1948 | 4 | *b *c | <0.001 |
|  |  | 4 |  |  | 4 |  |  | 4 |  |  |
| Human activities are contributing to climate change. | 242 | 4.7 | ***a ***b | 1380 | 3.2 | ***a ***c | 1964 | 3.5 | ***b ***c | <0.001 |
|  |  | 5 |  |  | 3 |  |  | 4 |  |  |
| Climate change will not affect the way that the [stakeholders] [operate/lives]. | 242 | 1.7 | ***a ***b | 1381 | 2.6 | ***a ***c | 1965 | 2.2 | ***b ***c | <0.001 |
|  |  | 1.5 |  |  | 3 |  |  | 2 |  |  |
| There is enough evidence that climate is changing. | 242 | 4.4 | ***a ***b | 1383 | 3.2 | ***a ***c | 1966 | 3.5 | ***b ***c | <0.001 |
|  |  | 5 |  |  | 3 |  |  | 4 |  |  |
| Notes. Survey questions: “In your opinion, what would the majority of [stakeholder’s] level of agreement be with the following statements?” and “Please indicate your level of agreement with the following statements.” The statement that asked about the scientists’ own perspective was “Climate change will not affect the way that I live.”  scale: 1 = strongly disagree, 2 = disagree, 3 = neither agree nor disagree, 4 = agree, 5 = strongly agree  "p" column indicates the statistical significance of the pairwise comparison (a, b, c) determined by the Wilcoxon-Mann-Whitney test (the alternative hypothesis is two-sided); *, **, and *** corresponds to a statistical significance level at 0.05, 0.01, and 0.001 respectively. The p-value column (Kruskal-Wallis test) indicates whether the distribution of the responses for all three groups were different by statement.  ^1^Surveys of producers (Singh et al. 2018) and crop advisors (Koundinya et al. 2017) were conducted as separate surveys in 2016. Only eight statements were used for comparison since two additional questions were developed post-distribution of the producer and crop advisor surveys. | | | | | | | | | | |
