# Supplementary material for: Understanding scientists’ communication challenges at the intersection of climate and agriculture
Source: PLoS One. 2022 Aug 2;17(8):e0269927. doi: 10.1371/journal.pone.0269927 (PMC9345487; doi:10.1371/journal.pone.0269927)
Supplement: S5 Table — (DOCX) [file pone.0269927.s008.docx]

**Table 5. Scientists’ likelihood of using terminology by stakeholder group sorted by overall decreasing likelihood ranking (mean)**

| **Term/phrase** | **Agribusinesses** | | | | **Crop advisors** | | | | **Producers** | | | | **Public** | | | | **Policymakers** | | | | **p-value** | **Overall** | | |
| --- | --- | --- | --- | --- | --- | --- | --- | --- | --- | --- | --- | --- | --- | --- | --- | --- | --- | --- | --- | --- | --- | --- | --- | --- |
|  | ***n*** | ***M*** | ***Mdn*** |  | ***n*** | ***M*** | ***Mdn*** |  | ***n*** | ***M*** | ***Mdn*** |  | ***n*** | ***M*** | ***Mdn*** |  | ***n*** | ***M*** | ***Mdn*** |  |  | ***n*** | ***M*** | ***Mdn*** |
| Extreme weather | 20 | 4 | 4 |  | 10 | 4.5 | 5 |  | 74 | 4.4 | 5 |  | 70 | 4.3 | 4.5 |  | 60 | 4.3 | 4 |  | 0.5943 | 234 | 4.4 | 5 |
| Sustainability | 20 | 4.7 | 5 | *a  **b | 10 | 4.4 | 4 |  | 73 | 3.9 | 4 | *a | 70 | 3.9 | 4 | **b | 60 | 4 | 4 |  | 0.0062* | 233 | 4 | 4 |
| Weather variability | 20 | 4.2 | 4 |  | 10 | 4.4 | 4.5 |  | 74 | 4.2 | 4 |  | 69 | 3.9 | 4 |  | 60 | 3.8 | 4 |  | 0.0519 | 233 | 4 | 4 |
| Variability | 19 | 4.1 | 4 |  | 10 | 4.4 | 4.5 |  | 73 | 4 | 4 |  | 70 | 3.9 | 4 |  | 59 | 4 | 4 |  | 0.6245 | 231 | 4 | 4 |
| Climate variability | 20 | 3.8 | 4 |  | 10 | 4.3 | 5 |  | 74 | 3.9 | 4 |  | 70 | 3.9 | 4 |  | 60 | 4.1 | 4 |  | 0.4218 | 234 | 4 | 4 |
| Resiliency | 20 | 4.2 | 4 |  | 10 | 4.3 | 4 |  | 73 | 3.7 | 4 |  | 68 | 3.9 | 4 |  | 60 | 4.2 | 4 |  | 0.0461* | 231 | 4 | 4 |
| Climate change | 20 | 3.9 | 4 |  | 10 | 3.9 | 4 |  | 74 | 3.6 | 4 | **a  **b | 70 | 4.2 | 4 | **a | 60 | 4.2 | 4 | **b | 0.0028* | 234 | 3.9 | 4 |
| Long-term weather | 20 | 4.2 | 4 |  | 10 | 4.5 | 4.5 |  | 73 | 3.9 | 4 |  | 70 | 3.7 | 4 |  | 60 | 3.7 | 4 |  | 0.0912 | 233 | 3.8 | 4 |
| Stress | 20 | 4 | 4 |  | 10 | 4.3 | 4 |  | 72 | 3.9 | 4 |  | 68 | 3.6 | 4 |  | 60 | 3.9 | 4 |  | 0.0480* | 230 | 3.8 | 4 |
| Unusual weather | 20 | 3.6 | 4 |  | 10 | 4.3 | 4.5 |  | 73 | 3.8 | 4 |  | 70 | 3.7 | 4 |  | 60 | 3.6 | 4 |  | 0.2400 | 233 | 3.7 | 4 |
| Greenhouse gases | 20 | 4.2 | 4 |  | 9 | 3.8 | 4 |  | 73 | 3.4 | 4 |  | 70 | 3.9 | 4 |  | 60 | 3.8 | 4 |  | 0.0273* | 232 | 3.7 | 4 |
| Rapid change | 20 | 3.7 | 4 |  | 10 | 3.9 | 4 |  | 73 | 3.4 | 4 |  | 68 | 3.8 | 4 |  | 60 | 3.7 | 4 |  | 0.2016 | 231 | 3.6 | 4 |
| Manmade and/or human made | 20 | 4.1 | 4 | *a | 10 | 3.7 | 4 |  | 73 | 3.1 | 3 | *a  ***b  *c | 70 | 3.9 | 4 | ***b | 60 | 3.7 | 4 | *c | 0.0003* | 233 | 3.6 | 4 |
| Uncertainty | 20 | 3.3 | 3.5 |  | 10 | 3.2 | 3.5 |  | 73 | 3.1 | 3 | **a | 69 | 3.3 | 4 |  | 60 | 3.8 | 4 | **a | 0.0157* | 232 | 3.4 | 4 |
| Global warming | 20 | 4 | 4 | **a | 10 | 2.9 | 3 |  | 74 | 2.9 | 3 | **a | 70 | 3.4 | 4 |  | 60 | 3.3 | 3.5 |  | 0.0059* | 234 | 3.2 | 3 |
| Positive trend | 19 | 3.3 | 3 |  | 10 | 3.4 | 4 |  | 73 | 3.3 | 4 |  | 68 | 3.1 | 3 |  | 60 | 3.3 | 3.5 |  | 0.6314 | 230 | 3.2 | 3 |
| Negative trend | 20 | 3.4 | 3 |  | 10 | 3.4 | 4 |  | 73 | 3.2 | 3 |  | 68 | 3 | 3 |  | 60 | 3.2 | 3 |  | 0.6208 | 231 | 3.2 | 3 |
| Holistic approach | 19 | 3.7 | 4 |  | 10 | 3.2 | 3.5 |  | 73 | 3.1 | 3 |  | 69 | 2.9 | 3 |  | 59 | 3.2 | 3 |  | 0.0924 | 230 | 3.1 | 3 |
| Social-ecological systems | 20 | 3.4 | 3 |  | 10 | 3.2 | 3 |  | 73 | 2.6 | 2 | **a | 68 | 2.9 | 3 |  | 60 | 3.4 | 3 | **a | 0.0057* | 231 | 3 | 3 |
| Green development | 19 | 3.1 | 3 |  | 10 | 2.8 | 3 |  | 74 | 2.4 | 2 | *a  *b | 70 | 3.1 | 3 | *a | 60 | 3.1 | 3 | *b | 0.0040* | 233 | 2.8 | 3 |
| Theory | 20 | 3.2 | 3 |  | 10 | 3 | 3.5 |  | 72 | 2.5 | 2 |  | 70 | 2.7 | 3 |  | 60 | 2.9 | 3 |  | 0.0445 | 232 | 2.8 | 3 |
| Heterogeneity | 20 | 3 | 3 |  | 10 | 2.5 | 2 |  | 73 | 2.5 | 2 |  | 70 | 2.7 | 3 |  | 60 | 2.8 | 3 |  | 0.4171 | 233 | 2.7 | 3 |
| Error | 19 | 3 | 3 |  | 10 | 2.9 | 3 |  | 73 | 2.5 | 2 |  | 69 | 2.5 | 3 |  | 57 | 2.8 | 3 |  | 0.2539 | 228 | 2.6 | 3 |
| Climate debate | 20 | 2.5 | 2 |  | 10 | 2.8 | 2.5 |  | 71 | 2.2 | 2 |  | 68 | 2.6 | 2 |  | 60 | 2.6 | 3 |  | 0.1889 | 229 | 2.4 | 2 |
| Notes. Survey question: “How likely are you to use the following terms/phrases when talking to [stakeholder] about climate change?”  scale: 1 = extremely unlikely, 2 = unlikely, 3 = neither agree nor disagree, 4 = likely, 5 = extremely likely  a, b, c indicates the pairwise comparison which were determined by the Wilcoxon-Mann-Whitney test adjusted by Bonferroni correction method; *, **, and *** corresponds to a statistical significance level at 0.05, 0.01, and 0.001 respectively. | | | | | | | | | | | | | | | | | | | | | | | | |
| p-value (Kruskal-Wallis test) indicates significance (* = <0.05) across stakeholders for each term/phrase | | | | | | | | | | | | | | | | | | | | | | | | |
