# Supplementary material for: Understanding scientists’ communication challenges at the intersection of climate and agriculture
Source: PLoS One. 2022 Aug 2;17(8):e0269927. doi: 10.1371/journal.pone.0269927 (PMC9345487; doi:10.1371/journal.pone.0269927)
Supplement: S6 Table — (DOCX) [file pone.0269927.s009.docx]

**Table 6. Communication challenges codes and examples (N = 227)**

| **Code** | **Example** |
| --- | --- |
| Access to stakeholder | *"I see the fundamental challenge is the limited number of opportunities for scientists and stakeholders to interact and share information."* |
| Collective strategy | *“The issue is complicated by past approaches that look at climate change issues in isolation from the multiple drivers of change with which it interacts to shape livelihood sustainability.”* |
| Culture | *“The public in general only accepts science when it is convenient and fit their own beliefs.”* |
| Disinformation | *“Misinformation and (worse) disinformation being disseminated by various outlets.”* |
| Economics | *“The economic questions/issues associated with mitigation for climate change.”* |
| Fear of regulation | *“Fear of added regulation.”* |
| Knowledge gap | *“Need rigorous economic analysis of management options.”* |
| Making it personal | *“A challenge is the ability to tell them what is the impact on them and their future family members by climate change that is and will happen in the future. Make it personal and not global. Also, talk about the changes that they have already made in relation to changing climate.”* |
| None | *“Observational data is noisy. However, the crop producers that I work with know that the climate is changing and they are concerned. Communication is not a problem."* |
| Overworked | *“Many crop advisors especially University Extension have limited time and resources and they are stretched too thin. Funding to support more extension personnel will allow them to engage with their stakeholders and have the time to learn the latest on climate change.”* |
| Pathway of information | *“The methods of communication are difficult with extension agents using a variety of pathways that can be diverse. Some are very tech-savvy and rely in mobile phone communications and email. Others prefer to receive information face to face or in meetings. Meeting these demands can be challenging.”* |
| Pipeline to right people | *“Reaching the right people that can reach the greatest number of shareholders.”* |
| Politics | *“The need to disengage the audience's political filters before communication can be successful.”* |
| Risk management | *“Risk management.”* |
| Scientist knowledge | *“My lack of knowledge about the economic constraints of producers.”* |
| Stakeholder knowledge | *“The fundamental issue is the lack of knowledge of climate in general and climate change in particular on the part of the audience.”* |
| Time management | *“Having enough time to work with them to go through a complete decision making process while also attending to professional requirements of being a federal scientist (i.e., publishing).”* |
| Timescale | *“Getting them to think about long term changes in climate and how to adapt to them now.”* |
| Trust | *“Getting past, in some audiences, the initial distrust (with no real reason) of 'science' and the confusion between science and religion.”* |
| Uncertainty | *“Uncertainties regarding model projections.”* |
| Using layperson language | *“To present findings in a layman language understandable to general public.”* |
| Variability | *“Distinguishing between year to year variability and longer term trends.”* |

Notes. Survey question: “In your opinion, what is the fundamental challenge in communicating climate change issues to [stakeholder]?”
